# Evaluation of ecological and health risks of trace elements in soils of olive orchards and apportionment of their sources using the APCS-MLR receptor model

**DOI:** 10.1007/s10653-024-02108-x

**Published:** 2024-07-16

**Authors:** Aişe Deliboran, Memet Varol, Halil Aytop

**Affiliations:** 1 Olive Research Institute, İzmir, Turkey; 2 Faculty of Agriculture, Malatya Turgut Özal University, Malatya, Turkey; 3 Kahramanmaraş East Mediterranean Transitional Zone Agricultural Research of Institute, Kahramanmaraş, Turkey

**Keywords:** İzmir, Soil contamination indices, Risk assessment, Source identification

## Abstract

İzmir, Turkey’s third most populous city, is in an important position in terms of both agriculture and industry. The province, which contributes 9.3% to the country’s industrial production, also has an important potential in terms of olive cultivation. However, until now, no research has been undertaken to analyze the content of trace elements (TEs) in the soil of olive orchards in İzmir. This study was carried out to determine the pollution level and ecological risks of TEs in the olive orchards soils of İzmir province, to reveal their potential sources and to evaluate their health risks. Among the TEs, the average content of only Ni (37.9 mg/kg) exceeded the world soil average content (29 mg/kg), while the average content of only Cd (0.176 mg/kg) exceeded the upper continental crust content (0.09 mg/kg). Enrichment factor revealed that there was significant enrichment for Cd in 73.6%, Ni in 11.6% and Cr in 5.4% of olive orchards, respectively, due to polluted irrigation water and agrochemicals. Similarly, ecological risk factor indicated that there were moderate and considerable ecological risks for Cd in 48.8% and 23.3% of olive orchards, respectively. Absolute principal component scores-multiple linear regression (APCS-MLR) model showed that Ni and Cr in the study area are affected by agricultural sources, Al, Co, Cu, Fe, Mn, Pb and Zn originate from lithogenic sources, and Cd originates from mixed sources. Based on health risk evaluation methods, non-carcinogenic and carcinogenic effects would not be expected for residents. This study provides significant knowledge for evaluating soil TE pollution in olive orchards and serves a model for source apportionment and human health risk evaluation of TEs in other agricultural regions.

**Supplementary Information:**

The online version contains supplementary material available at 10.1007/s10653-024-02108-x.

## Introduction

Trace elements (TEs), which comprise a small part of the earth’s crust, occur naturally in soils. Therefore, the contents of TEs in uncontaminated soils primarily depend on the parent materials (Aytop et al., 2023; Hu et al., 2020; Varol et al., 2021; Wang et al., 2021; Wei et al., 2023; Zinn et al., 2020). However, high TE contents in soils are associated with human activities (Antoniadis et al., 2022; Sun et al., 2021; Wang et al., 2022). The contamination of soil, which is a critical component of human, animal and plant life, by TEs as a result of increasing urbanization, industrialization and intensive use of agrochemicals has turned into a global problem (Wei et al., 2023; Yao et al., 2023). High TE levels in agricultural areas deteriorate soil structure and function, reducing product quality or yield (Antoniadis et al., 2022; Guo et al., 2021; Wei et al., 2023). On the other hand, TEs taken from the soil by plant roots can pose a risk to human and animal health through the food chain as they are transported to the edible parts of plants (Fei et al., 2019; Wei et al., 2023; Yao et al., 2023).

Since soils play an important role in the general TE cycle in nature, contaminated soils can also be a source of various TEs (Sun et al., 2021; Varol et al., 2021). For example, excess TEs accumulating in agricultural areas can pollute surface water and groundwater resources. Furthermore, TEs may adversely affect human health through the soil-food chain and through inhalation, dermal contact and ingestion pathways (Bayraklı et al., 2023; Guo et al., 2021; Varol et al., 2021, 2022). Previous studies have reported that health problems such as cardiovascular problems, liver and kidney dysfunctions, hematological diseases, reproductive disorders, neurological dysfunctions, developmental disorders and cancer may occur in people exposed to high concentrations of TEs (Lin et al., 2023; Sun et al., 2021; Timofeev et al., 2019; Varol et al., 2021). In this regard, it is critical to conduct investigations to evaluate the health risks of TEs in agricultural soils.

Removing TEs from contaminated agricultural soils is extremely difficult, expensive and time consuming. The best strategy to combat TE pollution in agricultural soils is to prevent soil contamination with TEs (Akbay et al., 2022; Guo et al., 2021; Varol et al., 2021). Therefore, it is very important to reveal the sources and pollution status of TEs, and to determine the ecological and human health risks, in terms of both preventing agricultural soil pollution and providing useful data for making critical decisions about soil pollution management (Aytop et al., 2023; Aytop 2022; Wang et al., 2022). For identifying the pollution sources of TEs in agricultural soils and quantifying contribution rate of each pollution source, various receptor models such as chemical mass balance (CMB), positive matrix factorization (PMF) and absolute principal component scores-multiple linear regression (APCS-MLR) are utilized. APCS-MLR has the advantage that it does not require prior knowledge of the number of pollution sources and the corresponding source profiles, so it is widely used in source apportionment of TEs in soil, sediment and surface water (Qu et al., 2018; Xue et al., 2023; Zhang et al., 2021a). In addition, pollution indices like geoaccumulation index (*Igeo*), Nemerow pollution index (NPI) and enrichment factor (EF) are implemented to determine the pollution level of TEs in soils (Fei et al., 2019; Guo et al., 2021; Radomirović et al., 2021; Wang et al., 2023). Similarly, ecological risk indices such as Nemerow risk index (NRI) and ecological risk factor (Er) are implemented to evaluate the impact of TEs on the soil ecosystem (Radomirović et al., 2020; Xue et al., 2023; Zhang et al., 2021b). Health risk assessment methods play an important role in estimating the risks that may occur in humans exposed to TEs in agricultural soils through ingestion, dermal and inhalation (Rinklebe et al., 2019; Wang et al., 2023; Zhang et al., 2021b). Given the specificity of each index and method, it would be more appropriate to make a comparative assessment of them to accurately determine the pollution status of a particular region (Fei et al., 2019).

İzmir, one of Turkey’s most important industrial cities, is located in the Aegean region. There are thirteen organized industrial zones, four technology development zones and two free zones in the province, which contributes 9.3% to the country’s industry. Furthermore, the province is a significant agricultural center. According to the number of olive trees, the Aegean region has the largest share of the country (75%) and the province of İzmir accounts for 23% of this share (Delıboran et al., 2022). Some olive orchards in the province are irrigated with groundwater, while others are irrigated with the Gediz River, which is heavily polluted by domestic and industrial wastewater discharges. In addition, pesticides and fertilizers are used extensively in the region. For these causes, the soils of the olive orchards may be polluted by TEs. Elevated TE concentrations in the soils of the study area may cause ecological risks, as well as risks to human health as a result of soil ingestion, inhalation and dermal contact. However, no research has been undertaken to date to detect the contents of TEs in the soils of olive orchards in İzmir and to evaluate the ecological and human health risks. In this respect, this investigation is very important in terms of filling these gaps.

Considering the above points, in this investigation, it was aimed to assess the pollution degree and potential ecological risks of the TEs in the soils of olive orchards using various indices, to quantify the potential sources of TEs using the APCS-MLR model, and to evaluate the health risks of TEs for adults and children. The findings of this research may provide important information for the control and management of soil contamination and the protection of human health.

## Materials and methods

### Study region

İzmir, located in the Aegean or Western Anatolia region of Turkey, is the third most populated city in Turkey in terms of people population (4,394,694 people). In addition, it is one of the most important centers of the Aegean region in terms of industry and agriculture. Olive orchards constitute 28.1% of the 3450 km^2^ agricultural area, which corresponds to approximately 28.5% of the surface area (12,086 km^2^) of İzmir province (Özden et al., 2021). In addition, about 23% of the olive tree presence in the Aegean Region is in İzmir. Most of the olive orchards have clay-loam and sandy-loam soil structure. In addition, these soils, which are slightly alkaline, have low organic matter content. İzmir has Mediterranean climate characteristics. The average annual air temperature and precipitation values recorded in the province are 17.5 °C and 713.8 mm, respectively (Delıboran et al., 2022).

### Sampling of soils and analysis

**Fig. 1:**
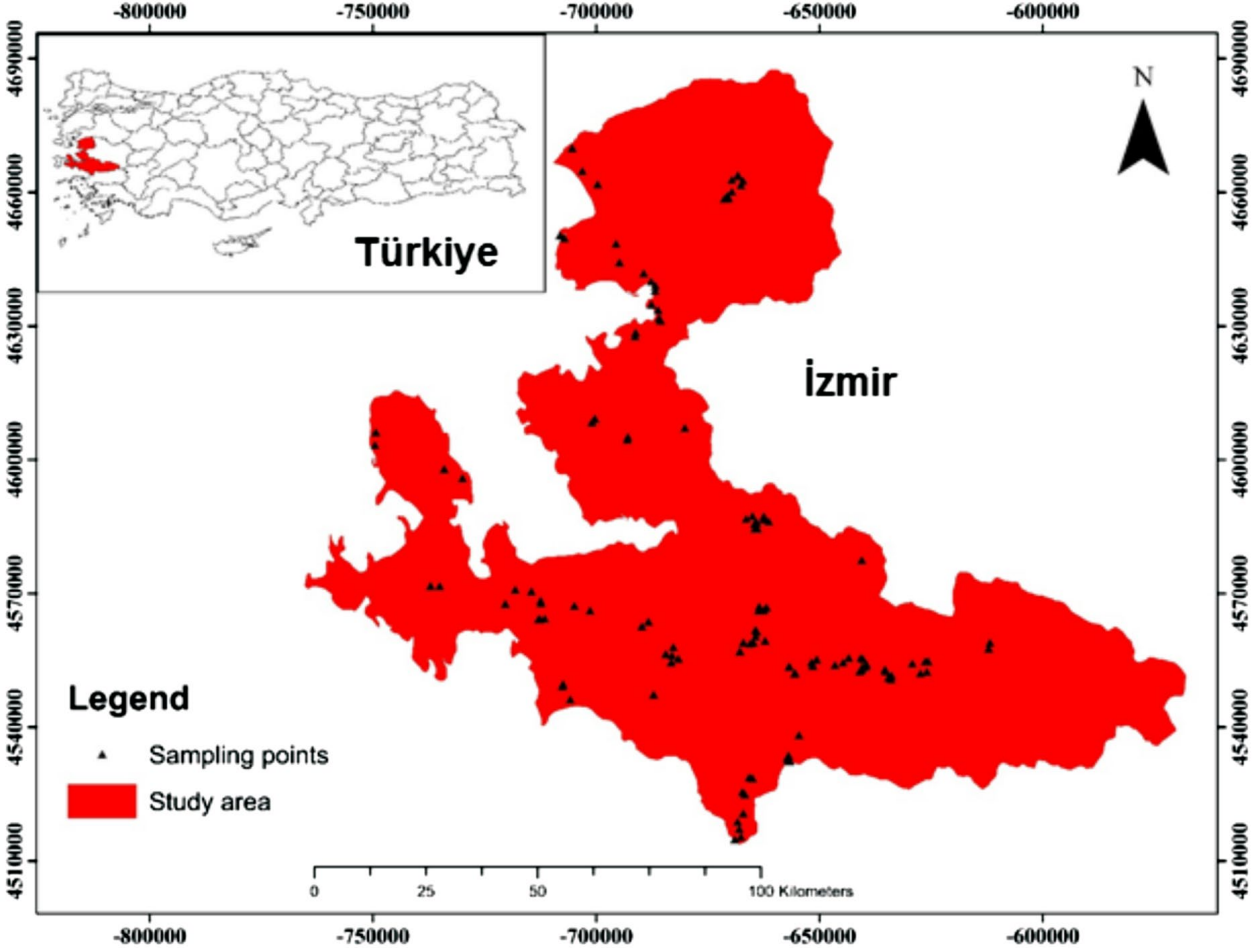
Map of İzmir province and sampling sites

Soil samples were taken from 0–30 cm depth from 129 olive orchards in İzmir in 2015. In this study, 8–10 individual samples were randomly collected from each orchard and mixed well to obtain a composite sample. Locations of 129 olive orchards were noted using the Global Positioning System (Fig. 1). The soils samples were delivered in nylon bags to the laboratory for TE analysis. They, which were first air-dried, were sieved using a 0.5 mm sieve, then ground into fine powder. Microwave digestion device (CEM MARS Xpress) was employed to digest the samples weighed in vessels with HNO_3_ and HCl (3:1) (Bayraklı et al., 2023; Lin et al., 2023). After cooling step, the digests were diluted to 50 mL volume with ultrapure water and filtered. The levels of 10 TEs (Al, Cd, Co, Cr, Cu, Fe, Mn, Ni, Pb and Zn) in the solutions were analyzed by Inductively Coupled Plasma-Optical Emission Spectroscopy (ICP-OES, Varian 720-ES) (Varol et al., 2021). Calibration standards were prepared by diluting a stock multi-element standard solution (1000 mg/L, Merck, Germany).

All reagents used for digestion and analysis in this investigation were of analytical grade. A variety of laboratory quality control and assurance techniques were employed to ensure analytical data quality, including analysis of blanks, replicates and a certified reference material (loamy clay, CRM052-050, RTC). The recoveries of TEs in the CRM ranged between 92.4% (Cd) and 108.4% (Ni) (Table S1). The analytical precision was within ± 10%. Analyses of each sample was performed in triplicate and mean values were used for data analysis. The results were expressed as mg/kg.

### Pollution levels and ecological risks of TEs

The pollution status of the soils in terms of TEs were determined through a combination of five different pollution indices (geoaccumulation index (*Igeo*), Nemerow pollution index (NPI), pollution load index (PLI), contamination factor (C_f_) and enrichment factor (EF)). The ecological risks of TEs in the soils were predicted using ecological risk factor (E_r_), Nemerow risk index (NRI) and ecological risk index (RI). Upper continental crust contents were used instead of background values of the TEs in the calculations of Cf, Igeo and EF (Delıboran et al., 2022). Details on these indices are given in Table 1. EF, Igeo, Cf, and Er indicate the pollution degree of a particular TE and its impact on the soil ecosystem (Hakanson, 1980; Müller, 1981). On the other hand, PLI, NPI, RI and NRI combine the effects of many TEs and indicate their overall pollution degree and their cumulative risk to the soil ecosystem (Hoang et al., 2021; Men et al., 2020).

**Table 1:** Pollution and ecological risk assessment indices used in this study

Indices	Equations	Explanations	Contamination or risk degree
*Pollution indices*			
Geoaccumulation index (I_geo_)	$Igeo = {\text{log}}_{2}\left[ {\frac{{{\text{Ci}}}}{{1.5 \times {\text{Bi}}}}} \right]$	B_i_ is background value of element (i); C_i_ is the content of element (i) (Müller, 1981)	I_geo_ ≤ 0 → Unpolluted 0 < I_geo_ < 1 → Unpolluted to moderately polluted 1 < I_geo_ < 2 → Moderately polluted 2 < I_geo_ < 3 → Moderately to heavily polluted 3 < I_geo_ < 4 → Heavily polluted 4 < I_geo_ < 5 → Heavily to extremely polluted I_geo_ ≥ 5 → Extremely polluted
Contamination factor (Cf)	${\text{C}}_{{\text{f}}}^{{\text{i}}} = \frac{{{\text{C}}^{{\text{i}}} }}{{{\text{C}}_{{\text{n}}}^{{\text{i}}} }}$	C_n_^i^ is pre-industrial value of element (i); C^i^ is the content of element (i) (Hakanson, 1980)	CF < 1 → Low contamination 1 < CF < 3 → Moderate contamination 3 < CF < 6 → Considerable contamination CF ≥ 6 → Very high contamination
Enrichment factor (EF)	${\text{EF}} = \left[ {\frac{{{\text{Ci}}}}{{{\text{Cref}}}}} \right]{\text{sample}}/\left[ {\frac{{{\text{Ci}}}}{{{\text{Cref}}}}} \right]{\text{background}}$	C_i_ is the content of element (i); C_ref_ is the content of reference element (Al) for geochemical normalization (Aytop et al., 2023)	EF < 2 → Minimal enrichment 2 ≤ EF < 5 → Moderate enrichment 5 ≤ EF < 20 → Significant enrichment 20 ≤ EF < 40 → Very high enrichment EF ≥ 40 → Extremely high enrichment
Pollution load index (PLI)	${\text{PLI}} = \left( {{\text{Cf}}_{1} \times {\text{Cf}}_{2} \times \ldots \times {\text{Cf}_{n}}} \right)^{1/n}$	Cf is the contamination factor value of each element; n is the number of elements (n = 10 in this study) Hoang et al., (2021)	PLI = 0 → No contamination 0 < PLI < 1 → Baseline contamination PLI > 1 → High contamination
Nemerow pollution index (NPI)	${\text{NPI}} = \sqrt {\frac{{\left( {{\text{Cf}_{mean}}} \right)^2{ } + { }\left( {{\text{Cf}_{max}}} \right)^2{ }}}{2}}$	Cf_mean_ and Cf_max_ are the mean and maximum value of the C_f_^i^ of all the elements studied Men et al., (2020)	NPI ≤ 0.7 → Unpolluted 0.7 < NPI ≤ 1 → Warning line of pollution 1 < NPI ≤ 2 → Low polluted 2 < NPI ≤ 3 → Moderately polluted NPI > 3 → Strongly polluted
*Ecological risk indices*			
Ecological risk factor (E_r_)	${\text{E}}_{{\text{r}}}^{{\text{i}}} = {\text{T}}_{{\text{r}}}^{{\text{i}}} \times {\text{C}}_{{\text{f}}}^{{\text{i}}}$	T_r_^i^ is the toxicity response coefficient of element (i), they are 1, 5, 30, 5, 2 and 5 for Zn, Cu, Cd, Ni, Cr and Pb, respectively. C_f_^i^ is the contamination factor of element (i) Hakanson, (1980)	E_r_ < 40 → Low potential ecological risk 40 ≤ E_r_ < 80 → Moderate potential ecological risk 80 ≤ E_r_ < 160 → Considerable potential ecological risk 160 ≤ E_r_ < 320 → High potential ecological risk E_r_ ≥ 320 → Very high potential ecological risk
Ecological risk index (RI)	${\text{RI}} = \mathop \sum \limits_{i = 1}^{n} {\text{E}}_{{\text{r}}}^{{\text{i}}} { }$	E_r_^i^ is the ecological risk factor of element (i); *n* i the number of studied elements (n = 6 in this study) Hakanson, (1980)	RI < 150 → Low ecological risk 150 ≤ RI < 300 → Moderate ecological risk 300 ≤ RI < 600 → Considerable ecological risk RI ≥ 600 → Very high ecological risk
Nemerow risk index (NRI)	${\text{NRI}} = \sqrt {\frac{{\left( {{\text{Er}_{mean}}} \right)^2{ } + { }\left( {{\text{Er}_{max}}} \right)^2{ }}}{2}}$	Er_mean_ and Er_max_ are the mean and maximum value of E_r_^i^ Men et al., (2020)	NRI ≤ 40 → Low risk 40 < NRI ≤ 80 → Moderate risk 80 < NRI ≤ 160 → Considerable risk 160 < NRI ≤ 320 → High risk NRI > 320 → Very high risk

### Health risk evaluation

Humans are mainly exposed to TEs in soil mostly through dermal contact, ingestion and inhalation routes. Therefore, in this investigation, health risks from exposure to TEs through these three routes were evaluated separately for residential adults and children (USEPA, 2023a). Hazard quotients (HQs) of TEs were calculated to evalute non-carcinogenic health risks. Due to the lack of carcinogenic slope or inhalation unit risk factors of other TEs, carcinogenic risks (CRs) were computed only for Cr, Cd, Ni and Co. The HQs and CRs of the TEs were estimated using the equations below (USEPA, 2023b). Tables S2 and S3 in the Supplementary File provided detailed explanations of these equations.

#### Non-carcinogenic risks

1 $${\text{HQingestion}} = { }\frac{{{\text{C}}s{ } \times {\text{ IRS}} \times {\text{RBA}} \times {\text{ EF }} \times {\text{ ED}} { }}}{{{\text{BW }} \times {\text{ AT}} \times {\text{ RfD}}o \times 10^6 }}$$

2 $${\text{HQdermal }} = { }\frac{{{\text{C}}s{ } \times {\text{SA}} \times {\text{AF}} \times {\text{ABS}}d \times {\text{EF }} \times {\text{ ED}}}}{{{\text{BW }} \times {\text{ AT}} \times {\text{ RfD}}o \times {\text{GIABS }} \times 10^6 }}$$

3 $${\text{HQinhalation }} = { }\frac{{{\text{C}}s{ } \times {\text{EF }} \times {\text{ ED}}}}{{{\text{AT}} \times {\text{ RfC }} \times {\text{ PEF}} }}$$

#### Carcinogenic risks

4 $${\text{CRingestion }} = \frac{{{\text{C}}s{ } \times {\text{ IFS }} \times {\text{RBA}} \times {\text{CSF}}o}}{{{\text{AT }} \times { }10^6}}$$

where: $${\text{IFS }} = \frac{{{\text{EF}} \times {\text{ ED}}a{ } \times {\text{ IRS}}a}}{{{\text{BW}}a}} + \frac{{{\text{EF}} \times {\text{ ED}}c{ } \times {\text{ IRS}}c}}{{{\text{BW}}c}}$$

5 $${\text{CRdermal }} = { }\frac{{{\text{C}}s{ } \times {\text{DFS}} \times {\text{ ABS}}d{ } \times {\text{CSF}}o}}{{{\text{AT}} \times {\text{GIABS }} \times 10^6 }}$$

where: $${\text{DFS }} = { }\frac{{{\text{EF }} \times {\text{ ED}}a \times {\text{SA}}a \times {\text{AF}}a}}{{{\text{BW}}a }} + \frac{{{\text{EF }} \times {\text{ ED}}c \times {\text{SA}}c \times {\text{AF}}c}}{{{\text{BW}}c}}$$

6 $${\text{CRinhalation }} = { }\frac{{{\text{C}}s{ } \times {\text{EF}} \times {\text{ED}} \times {\text{IUR}} \times 1000}}{{{\text{AT}} \times {\text{PEF}}}}$$

In this investigation, cumulative hazard quotient (CHQ), hazard index (HI), total HI (THI), cumulative carcinogenic risk (CCR), total carcinogenic risk (TCR) and cumulative TCR (CTCR) were also determined using the following equations:

7 $${\text{CHQ }} = { }\sum {\text{HQs of TEs}}$$

8 $${\text{HI }} = {\text{ HQingestion}} + {\text{HQdermal}} + {\text{HQinhalation }}$$

9 $${\text{THI }} = {\text{CHQing}} + {\text{CHQder}} + {\text{CHQinh }}or {\text{THI}} = \sum {\text{HIs of TEs}}$$

10 $${\text{CCR }} = { }\sum {\text{CRs of carcinogenic TEs}}$$

11 $${\text{TCR }} = {\text{ CRingestion}} + {\text{CRdermal}} + {\text{CRinhalation}}$$

12 $${\text{CTCR}} = {\text{CCRing}} + {\text{CCRder}} + {\text{CCRinh or}} {\text{CTCR}} = \sum {\text{TCRs of carcinogenic TEs}}$$

### Receptor model

The APCS-MLR receptor model is a widely used source apportionment technique to determine pollution sources of TEs in the soils and to compute the contributions (%) of pollution sources to each TE. Two different statistical methods including multiple linear regression (MLR) and absolute principal component scores (APCS) are used in this model. In the MLR analysis, TE contents were considered as the dependent variables, and APCS as the independent variables. The sources contributing to the concentration of each TE (Cj) can be determined by MLR as follows (Li et al., 2022; Xie et al., 2023):

$$C_{j} = b_{j} + \mathop \sum \limits_{h = 1}^{n} r_{hj} \times APCS_{hj}$$

where *b*_*j*_ is a multiple regression constant for parameter *j*; *r*_*hj*_ is a multiple regression coefficient of the source *h* with respect to *j*; *APCS*_*hj*_ is the scaled value of the rotated *h* for the considered sample; and *r*_*hj*_ × *APCS*_*hj*_ represents the contribution of source *h* to *C*_*j*_.

In the APCS-MLR model, negative contributions may appear in the calculation process. Although such negative values are correct, they can lead to confusion in interpreting and analyzing the contributions different pollution sources, potentially affecting the accuracy and precision of source apportionment. To overcome this issue, some researchers proposed a new method to convert all negative percentages to positive quantities, thus representing the contributions of the corresponding sources (Zhang et al., 2021a).

### Statistical analyses

Shapiro–Wilk normality test was conducted to check the distribution of the data of the variables. Spearman correlation test was employed to reveal the relationships between the variables. Before applying the APCS-MLR model, principal component analysis/factor analysis (PCA/FA) was employed to determine the types and numbers of potential pollution sources of TEs. The appropriateness of the data set for PCA/FA was checked with Bartlett sphericity and Kaiser–Meyer–Olkin (KMO) tests. In this study, PCA/FA, APCS-MLR model and Shapiro–Wilk normality test were performed using SPSS 22, and Spearman correlation diagram was performed using Origin 2020b.

## Results and discussion

### Contents of TEs in soils

Descriptive statistics of toxic elements (TEs) in olive orchard soils of the study area are given in Table 2. The mean concentrations of Al, Cd, Co, Cr, Cu, Fe, Mn, Ni, Pb and Zn were 22,521, 0.176, 7.58, 44.9, 19.1, 15,821, 352, 37.9, 8.85 and, 34.9 mg/kg, respectively. According to the Shapiro–Wilk test results, all TEs except Al and Fe did not show the normal distribution. In this study, Ni (104.4%), Cr (81.3%), Co (53.7%), Pb (50.2%) and Cd (49.3%) had higher coefficients of variation (CVs) (Table 2), implying that the spatial distributions of these TEs are not homogeneous and that human activities may be responsible for their contents (Fei et al., 2019; Zhang et al., 2021c). However, Al (24.97%), Fe (31.7%) and Zn (33.8%) had lower CV values (Table 1), suggesting that these TEs originate from natural sources. The skewness values of Co, Ni and Cr were > 2, indicating that these TEs could be affected by human activities (Xie et al., 2023).

**Table 2:** Descriptive statistics of TEs in this investigation and comparison with various guidelines and other investigations (unit in mg/kg)

İzmir	Al	Cd	Co	Cr	Cu	Fe	Mn	Ni	Pb	Zn	References
Mean	22,521	0.176	7.58	44.9	19.1	15,821	352	37.9	8.85	34.9	
Median	22,466	0.170	6.83	33.8	17.4	15,875	326	24.8	8.61	32.8	
Standart deviation	5623	0.0874	4.07	36.5	7.98	5013	148	39.5	4.44	11.8	
Standart error	495	0.0077	0.36	3.21	0.702	441	13.1	3.48	0.39	1.04	This study
Minimum	10,956	0.02	2.06	9.28	6.59	5464	133.9	6.5	1.29	13.4	
Maximum	37,522	0.42	39.8	210.7	47.8	28,834	984	312	25.4	79.6	
CV (%)	24.97	49.3	53.7	81.3	41.8	31.7	42.1	104.4	50.2	33.8	
Skewness	0.266	0.43	4.22	2.731	1.24	0.242	1.437	3.792	0.969	1.16	
World soil average (WSA)	–	0.41	11.3	59.5	38.9	–	488	29	27	70	Kabata-Pendias 2010)
European soil average (ESA)	–	0.28	10.4	94.8	17.3	–	524	37	32	68.1	Kabata-Pendias 2010)
Maximum allowable concentration (MAC)	–	5	50	200	150	–	–	60	300	300	Kabata-Pendias 2010)
Upper continental crust (UCC)	81,500	0.09	17.3	92	28	39,200	774	47	17	67	Rudnick et al., 2003)
Canadian soil quality guidelines (CSQGs)	–	1.4	40	64	63	–	–	50	70	200	CCME, 2007)
*Other studies*											
Amik Plain, Türkiye	–	–	20.4	–	–	–	–	274	5.6	–	Karanlık et al., 2011)
Bursa, Türkiye	–	–	–	125	40	–	1667	158	81	477	Aydinalp & Marinova, 2003)
Thrace region, Türkiye	–	–	11	173	20	26,900	600	50	33	45	Coşkun et al., 2006)
Sinop, Türkiye	–	–	–	194.73	43.19	38,849	–	85.02	17.01	65.1	Baltas et al., 2020)
Malatya, Türkiye	27,524	0.244	12.6	59.9	36.4	21,195	475	70.9	14.2	67	Varol et al., 2021)
Harran Plain, Türkiye	42,692		16	85	27	37,505	679	89	10.6	68	Varol et al., 2020)
Çanakkale, Türkiye	–	1.75	–	102.2	46.63	–	–	117.6	68.85	–	Sungur & İşler, 2021)

The mean contents of TEs determined in this study were compared with the world (WSA) and European (ESA) soil average TE concentrations suggested by Kabata-Pendias (Kabata-Pendias 2010). The results indicated that only the mean Ni concentration exceeded the limit values (Table 2). On the other hand, among the TEs in this research, the mean and median concentrations of only Cd were recorded to exceed the UCC concentration suggested by Rudnick and Gao (Rudnick et al., 2003) (Table 2). The mean and median contents of all TEs were lower than the maximum allowable concentrations (MACs) of TEs in soils suggested by Kabata-Pendias (2010), while only the maximum concentrations of Cr (210.7 mg/kg) and Ni (312 mg/kg) were above the MACs (200 mg/kg for Cr and 60 mg/kg for Ni) (Table 2). All Cd, Co, Cu, Pb and Zn contents were below the Canadian soil quality guidelines (CSQGs) for agricultural soils (CCME, 2007). The maximum contents of Ni and Cr exceeded the CSQGs, while their mean and median contents were below the CSQGs.

We compared the mean TE concentrations in olive orchard soils in this study with those in different agricultural regions of Turkey (Table 2). The Co and Ni contents recorded in our study were lower than those in Amik Plain, while the Pb content was higher. The contents of all TEs recorded in this study were found to be comparable to or lower than those of the agricultural soils of Bursa, Sinop, Malatya and Çanakkale provinces, Thrace region and Harran Plain (Table 2). These comparison results revealed that anthropogenic activities and spatial heterogeneity in soil properties have an impact on the TE contents of agricultural soils in different regions (Varol et al., 2021).

### Pollution status of the TEs

EF, Igeo and Cf indices are used to measure enrichment level or pollution status of a particular element in the soil. In this investigation, the mean EF values of TEs in the study area were found to be 1 for Al, 1.49 for Fe, 7.18 for Cd, 1.59 for Co, 1.79 for Cr, 2.56 for Cu, 1.63 for Mn, 2.94 for Ni, 1.91 for Pb and 1.93 for Zn (Table S4). According to these mean EF results, Al, Fe, Co, Cr, Mn, Pb and Zn exhibited “minimal enrichment”, whereas Cr and Ni exhibited “moderate enrichment” and Cd exhibited “significant enrichment” in the soils of the olive orchards. Considering all samples collected in the study area, > 85% of Al, Fe, Co and Mn exhibited “minimal enrichment” (Table 3), while 77.5% and 17.1% of Cr, 35.7% and 59.7% of Cu, 50.4% and 37.98% of Ni, 60.5% and 37.2% of Pb and 62.8% and 36.4% of Zn exhibited “minimal enrichment” and “moderate enrichment”, respectively (Table 3). On the other hand, 73.6% of the olive orchards exhibited “significant enrichment”for Cd (Table 3).

**Table 3:** Percent distributions (%) of TEs in the olive orchards according to pollution and ecological risk index classes

A-Individual indices										
Class	Al	Cd	Co	Cr	Cu	Fe	Mn	Ni	Pb	Zn
1-Contamination factor (C_f_)										
Low contamination	100	15.5	98.4	91.5	87.6	100	98.4	80.6	95.3	98.4
Moderate contamination	0	69.0	1.6	8.5	12.4	0	1.6	15.5	4.7	1.6
Considerable contamination	0	15.5	0	0	0	0	0	3.1	0	0
Very high contamination	0	0.0	0	0	0	0	0	0.78	0	0
2-Enrichment factor (EF)										
Minimal enrichment	100	6.98	87.60	77.52	35.7	91.47	87.60	50.39	60.47	62.79
Moderate enrichment	0	18.60	11.63	17.05	59.7	8.53	12.40	37.98	37.21	36.43
Significant enrichment	0	73.64	0.78	5.43	4.7	0	0	11.63	2.33	0.78
Very high enrichment	0	0.78	0	0	0	0	0	0	0	0
3-Geoaccumulation index (Igeo)										
Unpolluted	100	34.88	99.22	96.12	97.67	100	100	86.82	99.22	100
Unpolluted to moderately polluted	0	49.61	0.78	3.88	2.33	0	0	9.3	0.78	0
Moderately polluted	0	15.5	0	0	0	0	0	3.1	0	0
Moderately to heavily polluted	0	0	0	0	0	0	0	0.78	0	0
4-Ecological risk factor (E_r_)										
Low potential ecological risk	–	27.9	–	100	100	–	–	100	100	100
Moderate potential ecological risk	–	48.8	–	0	0	–	–	0	0	0
Considerable potential ecological risk	–	23.3	–	0	0	–	–	0	0	0

B-Synergistic indices							
1-Pollution load index (PLI)		2-Nemerow pollution index (NPI)		3-Ecological risk index (RI)		4-Nemerow risk index (NRI)	
*No contamination*	0	*Unpolluted*	25.6	Low ecological risk	98.4	*Low risk*	46.5
*Baseline contamination*	99.2	*Warning line of pollution*	48.8	Moderate ecological risk	1.6	*Moderate risk*	50.4
*High contamination*	0.8	*Low polluted*	22.5	–		*Considerable risk*	3.1
–		*Moderately polluted*	3.1	–		–	

In this study, the mean Igeo value of only Cd was positive (0.16), showing that the soils in the research region were “unpolluted to moderately polluted” by Cd (Table S4). On the other hand, the mean Igeo values of other TEs were negative, showing that the soils were “unpolluted” by other TEs (Table S4). In this study, Cd exhibited “unpolluted”, “unpolluted to moderately polluted” and “moderately polluted” status in 34.9%, 49.6% and 15.5% of the olive orchards, respectively. However, other TEs exhibited “unpolluted” status in > 85% of the olive orchards (Table 3).

The mean Cf value of Cd was 1.96, indicating that the soils exhibited “moderate contamination” status for Cd (Table S4). On the other hand, the mean Cf values of other TEs were below 1, showing “low contamination” in the soils of the olive orchards (Table S4). In this research, Al, Fe, Co, Cr, Cu, Mn, Pb and Zn exhibited “low contamination” status in > 85% of the olive orchards (Table 3). However, 15.5%, 69% and 15.5% of Cd and 80.6%, 15.5% and 3.1% of Ni exhibited “low contamination”, “moderate contamination” and “considerable contamination”, respectively (Table 3).

PLI and NPI are employed to determine the overall pollution level of multiple TEs in the soil. In this research, the mean PLI value was 0.52 (Table S4). Similarly, PLI values at 99.2% of the olive orchards were < 1 (Table 3), showing that the soils in the research region had “low contamination”. According to the mean NPI value (1.53) (Table S4), the soils of olive orchards were “low polluted”. The NPI results indicated that 25.6%, 48.8% and 22.5% of the olive orchards were “unpolluted”, “warning life of pollution” and “low polluted”, respectively (Table 3).

The results of the individual pollution indices (EF, Igeo and Cf) were consistent with each other. Based on the results of these indices, Cd exhibited a moderate pollution in most of the olive orchards, while Ni and Cr exhibited low and moderate pollution. In contrast, other TEs exhibited low pollution. In the study region, agrochemicals are widely used to increase agricultural production (Delıboran et al., 2022; Özden et al., 2021). In addition, some orchards are irrigated with the water of the Gediz River. Therefore, these agricultural activities may be primarily responsible for the enrichment of Cd in the soils of some olive orchards. Similarly, the results of synergistic pollution indices (PLI and NPI) were consistent with each other and indicated that most of the olive orchards exhibited low pollution due to the combined pollution effect of all TEs.

### Ecological risk assessment

The mean Er values of TEs in the study area were estimated to be 4.03 for Ni, 2.6 for Pb, 0.52 for Zn, 58.8 for Cd, 0.98 for Cr and 3.41 for Cu (Table S4). Since the mean Er values of Ni, Pb, Zn, Cr and Cu did not exceed 40, the soils had “low ecological risk” for these TEs. However, the average Er value of Cd in the soils of olive orchards exhibited “moderate potential ecological risk”. Considering all the olive orchards in the research region, it was found that 100% of them had “low ecological risk” for Ni, Pb, Zn, Cr and Cu (Table 3). On the other hand, 27.9%, 48.3% and 23.3% of the olive orchards had “low ecological risk”, “moderate potential ecological risk” and “considerable potential ecological risk” for Cd, respectively, due to agricultural activities (Table 3).

RI and NRI are employed to determine the combined ecological risks of many TEs in the soil. In this study, the mean RI value (70.4) was below 150 (Table S4), showing “low ecological risk”, whereas the mean NRI value was between 40 and 80 (Table S4), showing “moderate risk” in the soils. In the research region, 100% of the olive orchards had “low ecological risk” based on the RI values, while 46.5% and 50.4% of the olive orchards had “low risk” and “moderate risk” based on the NRI values, respectively (Table 3). Cd was the element with the highest contribution to the RI (83.6%) that agrees with the findings of a previous study (Zhang et al., 2020a). However, contribution rates of other TEs to the RI were below 6%. Similarly, Cd had the maximum Er values at all olive orchards, indicating that Cd was the major toxic element contributing to the NRI. As a result, due to Cd enrichment as a result of agricultural activities, almost half of the orchards had a moderate ecological risk, and the other half had a low ecological risk.

### Quantitative source identification of TEs

Firstly, PCA/FA was performed to reveal the sources of TEs in the soils. The KMO and Bartlett’s test results (KMO = 0.655; *p* = 0.000) demonstrated the suitability of the data for PCA/FA (Fei et al., 2019; Varol et al., 2021). The PCA/FA produced three principal components (PCs), whose eigenvalues were > 1, explaining 77.2% of the total variance. PC1, PC2 and PC3 explained 29.8%, 23.8% and 23.6% of the total variance, respectively. In addition, the data in our study met the criterion (n ≥ m + 50, where n indicates the number of sampling sites and m shows the number of TEs) of APCS-MLR model (Proshad et al., 2022). This implies that PCA/FA results were reliable and could be used to calculate source contributions. Then, the APCS-MLR model was conducted to determine sources of TEs in the soils and to reveal the contribution (%) of each source to TEs. To find the accuracy of the model, the ratio between the mean predicted (P) and measured (M) results of the TEs was used. In this study, all M/P ratios were found to be 1 (Table 4). Furthermore, the squares of the correlation coefficients (R^2^) of all TEs in the receptor model were determined to be > 0.55 (Table 4). These findings showed that the model used in the source apportionment is reliable (Li et al., 2022; Xue et al., 2023; Zhang et al., 2021a).

**Table 4:** The results of APCS-MLR model used for source apportionment

TEs	Measured mean level (M)	Predicted mean level (P)	M/P	R^2^	Source contribution (%)		
					PC1	PC2	PC3
Al	22,521	22,521	1	0.692	4.71	66.36	28.93
Cd	0.176	0.176	1	0.567	4.47	41.88	53.65
Co	7.58	7.58	1	0.872	23.76	30.33	45.91
Cr	44.9	44.9	1	0.896	67.57	1.80	30.62
Cu	19.1	19.1	1	0.721	0.115	2.57	97.32
Fe	15,821	15,821	1	0.79	6.35	20.88	72.76
Mn	352	352	1	0.674	5.74	70.22	24.04
Ni	37.9	37.9	1	0.931	72.31	14.34	13.35
Pb	8.85	8.85	1	0.798	6.21	89.56	4.23
Zn	34.9	34.9	1	0.779	0.317	41.51	58.17

**Fig. 2:**
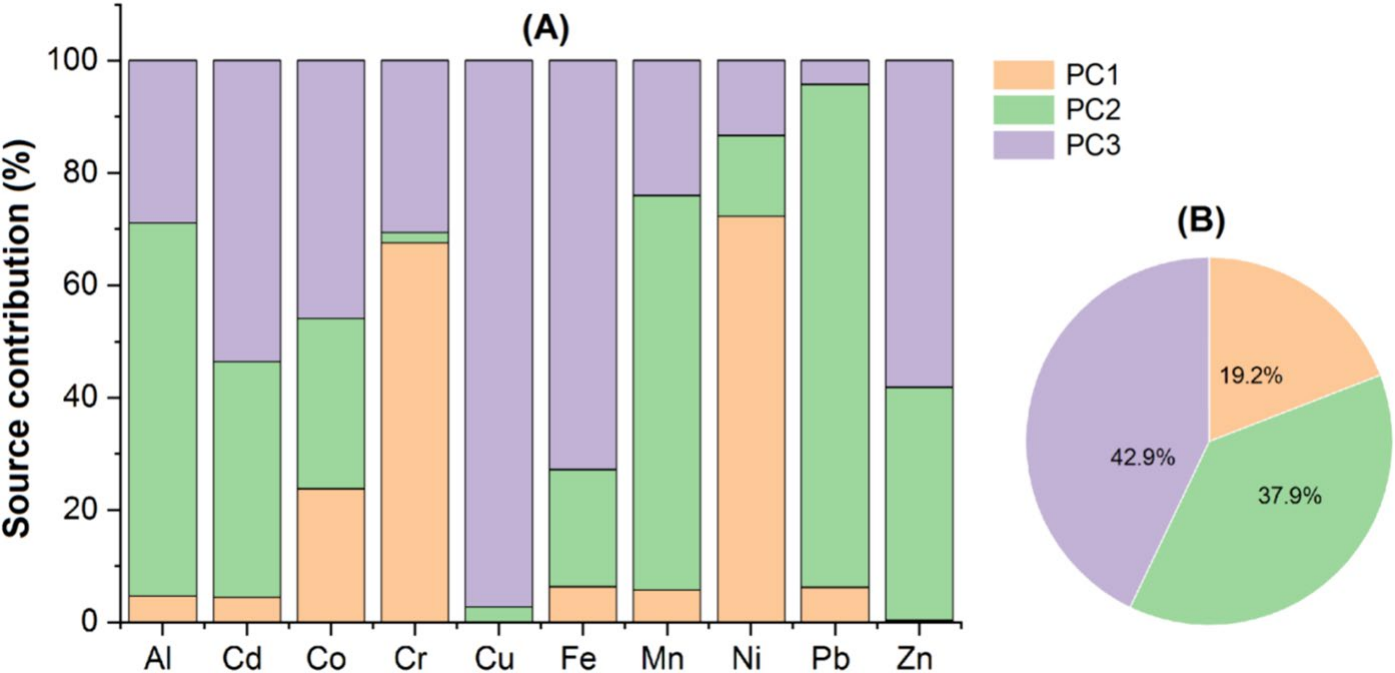
Contribution of different pollution sources to each element (**A**) and the mean contributions of sources (**B**) based on APCS-MLR receptor model

**Fig. 3:**
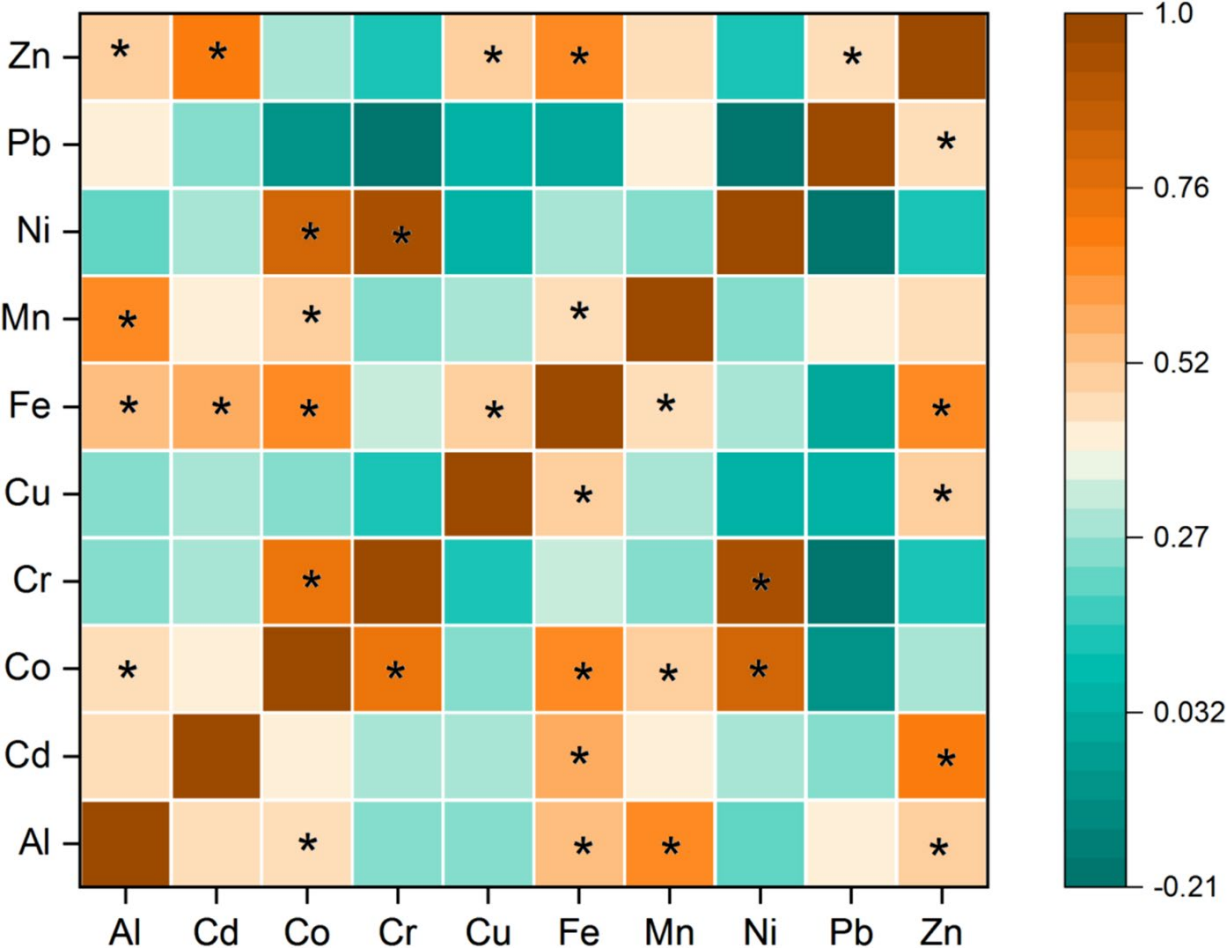
Spearman correlation matrix plot of elements (* r > 0.45; *p* < 0.01)

The PC1 accounted for 19.16% of the contribution rate and was mainly loaded with Ni (72.31%) and Cr (67.57%) (Fig. 2). In this study, both elements showed strong positive correlations (r = 0.951; *p* < 0.01) (Fig. 3), suggesting that they were from the similar sources. In addition, Ni and Cr had the highest CV and skewness values (Table 2), indicating that human activities may affect the spatial distributions of Cr and Ni. Likewise, it was observed that their maximum contents exceeded the CSQG and MAC values (Table 2). In general, high Cr and Ni contents in agricultural soils may be related with agrochemicals and contaminated irrigation water (Dinter et al., 2021; Nana et al., 2023; Soleimani et al., 2023; Varol et al., 2021). Irrigation of some olive orchards with the water of the Gediz River, which is seriously polluted due to domestic and industrial wastewater discharges, and the use of pesticides and fertilizers in the study area may have caused an increase in the Cr and Ni contents of the soils. These findings indicated that distributions of Ni and Cr in the study area are impacted by agricultural activities. Thus, PC1 was related to anthropogenic sources.

The PC2 had a contribution rate of 37.95% and showed high loadings on Pb (89.56%), Mn (70.22%) and Al (66.36%) (Fig. 2). Other studies reported that Al and Mn are lithogenic elements originating from natural sources, such as soil parent materials and rock weathering (Aytop et al., 2023; Varol et al., 2021; Zhang et al., 2020b). In this study, Al and Mn, which were correlated with each other (r = 0.67; *p* < 0.01) (Fig. 3), had low EF, Cf and Igeo values at almost of the olive orchards (Table 3). Furthermore, their mean and median values were lower than UCC values (Table 2). Therefore, these elements were primarily controlled by lithogenic sources. Similarly, the mean content of Pb was lower than UCC, WSA and MAC values (Table 2). In addition, it was significantly correlated with Al (r = 0.405; *p* < 0.01) (Fig. 3), indicating that they were from the same sources. The fact that Pb had low EF, Cf, Igeo and Er values confirmed this finding (Table 3). Thus, PC2 was mainly associated with lithogenic sources.

The PC3 accounted for 42.9% of the contribution rate and was mainly loaded with Cu (97.32%), Fe (72.76%), Zn (58.17%) and Cd (53.65%) and Co (45.91%) (Fig. 2). In this study, Fe, one of the most abundant metals in Earth’s crust (Varol et al., 2021), had low EF, Cf and Igeo values at most of the olive orchards (Table 3). In addition, its mean and median values were lower than UCC (Table 2). Therefore, Fe was primarily controlled by natural sources. Cu, Zn and Co in this component had positive correlations with Fe (r > 0.5; *p* < 0.01) (Fig. 3), showing that they came from the same origin. Their low EF, Cf and Igeo values (Table 4) and lower mean and median values of them than UCC, WSA and MAC values (Table 2) confirmed this finding. However, the mean and median contents of Cd were above the UCC value (Table 2). Furthermore, 73.6% and 69% of the olive orchards showed “significant enrichment” and “moderate contamination” for Cd, respectively. Similarly, 48.3% of the olive orchards had “moderate potential ecological risk” for Cd (Table 3). These findings indicated that anthropogenic activities may be an origin of Cd. According to the existing literature, phosphate fertilizers and contaminated irrigation water contribute to Cd enrichment in the agricultural soils (Fei et al., 2019; Han & Gu, 2023; Soleimani et al., 2023; Varol et al., 2021). On the other hand, the mean and median values of Cd were lower than WSA and MAC values (Table 2). In addition, it was correlated with Fe (r = 0.558; *p* < 0.01) (Fig. 3). Based on these findings, Cd originated from both natural and anthropogenic sources. Consequently, PC3 was associated with mixed sources (natural and agricultural sources).

### Health risk assessment

**Fig. 4:**
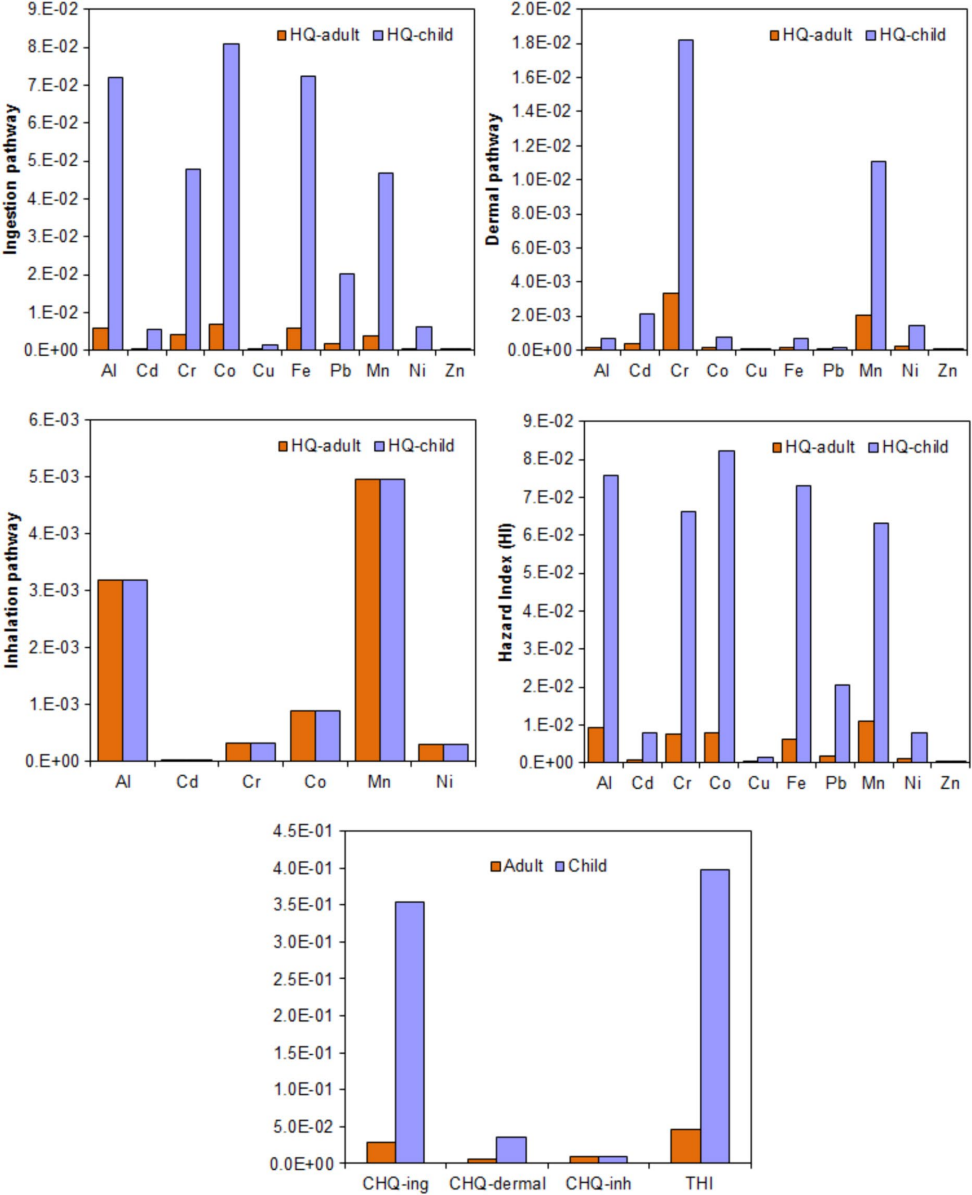
Non-carcinogenic health risks of TEs for residents via three routes of exposure (HQ, hazard quotient; CHQ, cumulative HQ; HI, hazard index; THI, total HI)

Non-carcinogenic (HQ, CHQ, HI and THI) and carcinogenic risk (CR, CCR, TCR and CTCR) results of TEs for residents were presented in Fig. 4 and Table 5. In this investigation, all HQ, CHQ, HI and THI values were below the acceptable level (namely 1.0) (Fig. 4), showing that non-carcinogenic risks due to exposure to TEs in the soils via inhalation, ingestion and dermal contact routes would not be expected for adults and children. Among the TEs, Co, Fe and Al for ingestion route, Cr, Mn and Cd for dermal route and Mn, Al and Co for inhalation route had the highest HQ values for residents.

**Table 5:** Carcinogenic health risks of TEs for residential receptors

	Carcinogenic risks			
	CR ingestion	CR dermal	CR inhalation	TCR
Cd	–	–	8.30E−11	8.30E−11
Cr	3.62E−05	1.53E−05	2.74E−06	5.43E−05
Co	–	–	1.79E−08	1.79E−08
Ni	–	–	2.58E−09	2.58E−09
	CCR	CCR	CCR	CTCR
	*3.62E−05*	*1.53E−05*	*2.76E−06*	*5.43E−05*

CR, carcinogenic risk; CCR, cumulative CR; TCR, total CR; CTCR, cumulative TCR

For both adults and children, the CHQ_ingestion_ values were higher than CHQ_inhalation_ and CHQ_dermal_ values (Fig. 4). The CHQ_ingestion_ values for adults and children contributed to 64.7% and 88.7% of the THI, respectively. According to these findings, it can be concluded that ingestion route had a greater negative impact on human health than dermal contact and inhalation routes. These findings were consistent with other studies (Wang et al., 2022; Wei et al., 2023; Zhang et al., 2021c). In addition, HI values of all TEs were higher in children than in adults. These results indicated that children are more affected by TEs, which is consistent with previous studies (Timofeev et al., 2019; Varol et al., 2021; Wang et al., 2023).

The CR values of Cd, Co and Ni for inhalation route were below the USEPA’s acceptable risk range of 1 × 10^−4^ and 1 × 10^−6^ (Table 5) In addition, the CR values of Cr for three routes and its TCR value were in this range. Similarly, all CCR values and CTCR values were in this range. These results indicated that carcinogenic risks due to exposure to Cr, Cd, Co and Ni in the soils of the olive orchards would not be expected for residents. In this research, the highest TCR value was determined for Cr (5.43E-05), followed by Co (1.79E-08), Ni (2.58E-09) and Cd (8.30E-11) (Table 5). The CCR_ingestion_ value was higher than the CCR_dermal_ and CCR_inhalation_ values, which is line with other studies. The CCR_ingestion_ value contributed to 66.7% of the CTCR value, while CCR_dermal_ and CCR_inhalation_ values contributed to 28.2% and 5.08% of the CTCR value, respectively. These findings revealed that the receptors are exposed to TEs in the soil primarily through ingestion route.

## Conclusions

The present research was conducted to determine the pollution degrees and ecological risks of TEs in the soils of olive orchards in İzmir, to determine their potential sources and to assess their health risks. The results showed that Ni and Cr had high CV and skewness values, indicating the effect of anthropogenic activities on the spatial distributions of both elements. Among the TEs, mean content of only Ni exceeded the WSA content, while mean content of only Cd exceeded the UCC content. However, mean contents of all TEs were below the MACs. The contents of all TEs determined in this study were found to be comparable to or lower than those of the agricultural soils located in different regions of Turkey. The EF and Cf results indicated that most of olive orchards had “significant enrichment” and “moderate contamination” for Cd. Similarly, 48.3% of the olive orchards had “moderate potential ecological risk” for Cd. However, based on the results of PLI, NPI and RI, low pollution and low ecological risk were recorded in the soils of the olive orchards. The APCS-MLR receptor model determined three potential sources in decreasing order of mixed sources (42.9%) > lithogenic sources (37.95%) > anthropogenic sources (19.16%). All non-carcinogenic risk values (HQ, CHQ, HI and THI) were below 1, showing that no adverse health effects would be expected for adults and children. The HQ and HI values of TEs were higher in children than in adults, suggesting that children are more affected by TEs in the soils of olive orchards. All carcinogenic risk values (CR, CCR, TCR and CTCR) were in or below the USEPA’s acceptable risk range, showing that carcinogenic effects would not be expected for residents.

This study provides significant knowledge for evaluating soil TE pollution in olive orchards and serves a model for source apportionment and human health risk evaluation of TEs in other agricultural regions. Also, further research is required to investigate the concentrations of TE in irrigation water and olives, and the contents of TEs in olive orchard soils should be monitored regularly.

## Supplementary Information

Below is the link to the electronic supplementary material.

Supplementary file1 (DOCX 42 kb)

## Data Availability

All data analyzed during this study are included in this published article and are not publicly available but may be obtained from the corresponding author on reasonable request.
