# Supplementary material for: Evaluation of ecological and health risks of trace elements in soils of olive orchards and apportionment of their sources using the APCS-MLR receptor model
Source: Environ Geochem Health. 2024 Jul 16;46(9):320. doi: 10.1007/s10653-024-02108-x (PMC11252231; doi:10.1007/s10653-024-02108-x)
Supplement: Supplementary file 1 — Supplementary file1 (DOCX 42 kb) [file 10653_2024_2108_MOESM1_ESM.docx]

**Table S1.** Concentrations (mg/kg) of TEs found in certified reference material (CRM052-050, RTC) and mean recoveries (%)

|  | Al | Cd | Co | Cr | Cu | Fe | Mn | Ni | Pb | Zn |
| --- | --- | --- | --- | --- | --- | --- | --- | --- | --- | --- |
| Reference value | 10900 | 35.6 | 26.3 | 30.7 | 44.2 | 12400 | 187 | 28.6 | 82.6 | 89 |
| Measured value (n=5) | 11045 | 32.9 | 28.1 | 32.4 | 43.9 | 12528 | 198 | 31 | 78.5 | 93.2 |
| Recovery (%) | 101.3 | 92.4 | 106.8 | 105.5 | 99.3 | 101.0 | 105.9 | 108.4 | 95.0 | 104.7 |

**Table S2.** Parameters used for health risk assessment of TEs in soil

| Parameters | Symbols | Units | Values | References |
| --- | --- | --- | --- | --- |
| Element concentration | C_s_ | mg/kg |  | Site-specific |
| Body weight-adult | BW_a_ | kg | 72 | Site-specific |
| Body weight-child | BW_c_ | kg | 15 | USEPA (1991) |
| Exposure duration-child | Ed_c_ | years | 6 | USEPA (1991) |
| Expose duration-adult | Ed_a_ | years | 20 | USEPA (2023a) |
| Exposure frequency | EF | days/year | 350 | USEPA (1991) |
| Skin surface area-adult | SA_a_ | cm^2^ | 6032 | USEPA (2011) |
| Skin surface area-child | Sa_c_ | cm^2^ | 2373 | USEPA (2011) |
| Soil intake ratio-adult | IRS_a_ | mg/day | 20 | Jia et al. (2018) |
| Soil intake ratio-child | IRS_c_ | mg/day | 50 | Jia et al. (2018) |
| Averaging time-child | AT_c_ | days | 365 x ED_c_ (non-carcinogenic) | USEPA (1989) |
| Averaging time-adult | AT_a_ | Days | 365 x ED_a_ (non-carcinogenic) | USEPA (1989) |
| Skin adherence factor-adult | AF_a_ | mg/cm^2^ | 0.07 | USEPA (2002) |
| Skin adherence factor-child | AF_c_ | mg/cm^2^ | 0.2 | USEPA (2002) |
| Soil ingestion ratio | IFS | mg/kg | Age-adjusted (8944.444) | USEPA (2023a) |
| Soil dermal contact factor | DFS | mg/kg | Age-adjusted (107495.111) | USEPA (2023a) |
| Life time | LT | Years | 70 | Site-specific |
| Averaging time | AT | Days | 365 x LT = 25550 (carcinogenic) | Site-specific |

**Table S3.** RBA, ABS_d_, RfD_o_, CSF_o_, GIABS, RFC, PEF and IUR values of TEs used for health risk assessment

| Element | Relative  bioavailability  factor (RBA)  (unitless) | Dermal absorption  fraction (ABS_d_)  (unitless) | Oral reference  dose (RfD_o_) (mg/kg-day) | Oral slope factor (CSF_o_)  (mg/kg-day)^-1^ | Gastrointestinal  Absorption (GIABS)  (unitless) | Inhalation reference  concentration (RFC) (mg/m^3^) | Particulate  emission factor  (PEF) (m^3^/kg) | Inhalation unit  risk (IUR) (µg/m^3^)^-1^ |
| --- | --- | --- | --- | --- | --- | --- | --- | --- |
| Al | 1 | 0.001 | 1 | - | 1 | 0.005 | 1.36 x 10^9^ | - |
| Cd | 1 | 0.001 | 0.0001 | - | 0.025 | 0.00001 | 1.36 x 10^9^ | 0.0018 |
| Co | 1 | 0.001 | 0.0003 | - | 1 | 0.000006 | 1.36 x 10^9^ | 0.009 |
| Cr | 1 | 0.001 | 0.003 | 0.5 | 0.025 | 0.0001 | 1.36 x 10^9^ | 0.084 |
| Cu | 1 | 0.001 | 0.04 | - | 1 | - | 1.36 x 10^9^ | - |
| Fe | 1 | 0.001 | 0.7 | - | 1 | - | 1.36 x 10^9^ | - |
| Mn | 1 | 0.001 | 0.024 | - | 0.04 | 0.00005 | 1.36 x 10^9^ | - |
| Ni | 1 | 0.001 | 0.02 | - | 0.04 | 0.00009 | 1.36 x 10^9^ | 0.00026 |
| Pb | 1 | 0.001 | 0.0014 | - | 1 | - | 1.36 x 10^9^ | - |
| Zn | 1 | 0.001 | 0.3 | - | 1 | - | 1.36 x 10^9^ | - |
| References | USEPA (2023a) | USEPA (2004) | USEPA (2023b) | USEPA (2023b) | USEPA (2023b)  Jia et al. (2018) | USEPA (2023b) | USEPA (2023b) | USEPA (2023b) |

**Table S4.** Mean, maximum and minimum values of pollution and ecological risk indices

|  | | **Al** | | **Cd** | **Co** | **Cr** | | **Cu** | **Fe** | **Mn** | **Ni** | **Pb** | **Zn** |
| --- | --- | --- | --- | --- | --- | --- | --- | --- | --- | --- | --- | --- | --- |
| **Individual indices** | | | |  |  |  | |  |  |  |  |  |  |
| EF | Mean | 1 | | 7.18 | 1.59 | 1.79 | | 2.56 | 1.49 | 1.63 | 2.94 | 1.91 | 1.93 |
|  | Max | 1 | | 27.40 | 5.81 | 10.09 | | 8.13 | 3.09 | 3.88 | 17.91 | 6.16 | 5.95 |
|  | Min | 1 | | 0.71 | 0.39 | 0.31 | | 0.89 | 0.69 | 0.88 | 0.42 | 0.26 | 0.98 |
|  |  |  | |  |  |  | |  |  |  |  |  |  |
| Igeo | Mean | -2.49 | | 0.16 | -1.92 | -1.92 | | -1.25 | -1.97 | -1.83 | -1.32 | -1.72 | -1.60 |
|  | Max | -1.70 | | 1.71 | 0.62 | 0.61 | | 0.19 | -1.03 | -0.24 | 2.14 | 0.16 | -0.34 |
|  | Min | -3.48 | | -2.96 | -3.65 | -3.89 | | -2.67 | -3.43 | -3.12 | -3.44 | -4.30 | -2.91 |
|  |  |  | |  |  |  | |  |  |  |  |  |  |
| Cf | Mean | 0.276 | | 1.961 | 0.438 | 0.488 | | 0.682 | 0.404 | 0.455 | 0.806 | 0.521 | 0.521 |
|  | Max | 0.460 | | 4.909 | 2.300 | 2.291 | | 1.708 | 0.736 | 1.271 | 6.627 | 1.671 | 1.188 |
|  | Min | 0.134 | | 0.193 | 0.119 | 0.101 | | 0.235 | 0.139 | 0.173 | 0.138 | 0.076 | 0.200 |
|  |  |  | |  |  |  | |  |  |  |  |  |  |
| Er | Mean | - | | 58.83 | - | 0.98 | | 3.41 | - | - | 4.03 | 2.60 | 0.52 |
|  | Max | - | | 147.3 | - | 4.58 | | 8.54 | - | - | 33.1 | 8.36 | 1.19 |
|  | Min | - | | 5.79 | - | 0.202 | | 1.18 | - | - | 0.69 | 0.38 | 0.20 |
|  |  |  | |  |  |  | |  |  |  |  |  |  |
| **Synergistic indices** | | | |  |  |  | |  |  |  |  |  |  |
|  | **PLI** | | |  | **NPI** | | |  | | **NRI** |  | | **RI** |
| Mean | 0.524 | |  |  | 1.531 | |  |  |  | 42.4 |  |  | 70.4 |
| Max | 1.115 | |  |  | 4.855 | |  |  |  | 105.8 |  |  | 158.3 |
| Min | 0.209 | |  |  | 0.359 | |  |  |  | 5.1 |  |  | 12 |

**References**

Jia, Z., Li, S., Wang, Li., 2018. Assessment of soil heavy metals for eco-environment and human health in a rapidly urbanization area of the upper Yangtze Basin. Sci. Rep. 8, 3256.

USEPA, 1989. Risk assessment guidance for Superfund. Volume I: Human health evaluation manual (Part A). Interim Final. Office of Emergency and Remedial Response. EPA/540/1-89/002.

USEPA, 1991. Human health evaluation manual, supplemental guidance: "Standard default exposure factors ". OSWER Directive 9285.6-03.

USEPA, 2002. Supplemental Guidance for Developing Soil Screening Levels for Superfund Sites. OSWER 9355.4-24.

USEPA, 2004. Risk Assessment Guidance for Superfund Volume I: Human Health Evaluation Manual (Part E, Supplemental Guidance for Dermal Risk Assessment) Final. OSWER 9285.7-02EP.July 2004.

USEPA, 2011. Exposure Factors Handbook 2011 Edition. National Center for Environmental Assessment, Office of Research and Development. Washington D.C.

USEPA, 2023a. Regional Screening Levels (RSLs) - User's Guide. https://www.epa.gov/risk/regional-screening-levels-rsls-users-guide, Accessed date: 08 June 2023.

USEPA, 2023b. Regional Screening Level (RSL) Summary Table (TR=1E-06 THQ=1.0). https://semspub.epa.gov/work/HQ/197414.pdf, Accessed date: 08 June 2023.
